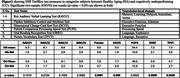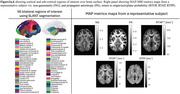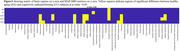# Gray matter microstructural changes due to cognitive aging in healthy cohort

**DOI:** 10.1002/alz.095565

**Published:** 2025-01-09

**Authors:** Kavita Singh, Stephanie Barsoum, Kurt Schilling, Yang An, Luigi Ferrucci, Dan Benjamini

**Affiliations:** ^1^ Multiscale Imaging and Integrative Biophysics Unit, National Institute on Aging, NIH,, Baltimore, MD USA; ^2^ Department of Radiology and Radiological Sciences, Vanderbilt University Medical Center,, Nashville, TN USA; ^3^ Brain Aging and Behavior Section, National Institute on Aging, NIH, Baltimore, MD USA; ^4^ Translational Gerontology Branch, National Institute on Aging, NIH, Baltimore, MD USA

## Abstract

**Background:**

Cognitive impairment with age remains undetected until it interferes daily life activity or presents dementia symptoms. In the US, 61% of dementia population is not diagnosed, which is in part due to limited sensitivity of clinical neuroimaging modalities in assessing early gray matter (GM) changes. Here we look at microstructural changes in GM using mean apparent propagator (MAP‐MRI) in cognitively underperforming (CU) and healthy aging (HA) cohorts, grouped according to their cognitive performance based on the NIH Toolbox.

**Methods:**

725 subjects aged 36 to 90 years were included in this study using the Human Connectome Project‐Aging (HCP‐A) imaging dataset (T1w, multi‐shell diffusion ‐dMRI). Nine NIH Toolbox measures were used to classify CU and HA population groups (**Figure 1**). Each test score was z‐normalized and subjects in the lower and upper 50^th^ percentile in all 9 tests were considered as CU and HA, respectively. Diffusion data was processed using TORTOISE dMRI processing package. Corrected DWIs were processed using MAP‐MRI, which yielded the following parameters: return to origin/axis/plane probability (RTOP, RTAP, RTPP), non‐Gaussianity (NG), and propagator anisotropy (PA) (**Figure 2a**). The T1w images were processed generating 125 SLANT cortical and subcortical regions of interest (**Figure 2b**). Two‐way ANOVA was used to investigate group differences while accounting for age, sex, education and site. All statistical analysis was done in Matlab2022b.

**Results:**

The list of tests and respective scores of the two groups is provided in Figure 1. Zero‐displacement probabilities were more sensitive in detecting group differences compared to NG and PA. The two groups displayed notable differences in the following brain regions: the operculum, involved in visuospatial cognition; the precuneus, key to memory retrieval; and the posterior cingulate, which plays a role in processing speed, executive function, and memory. Additionally, the auditory and sensory‐related areas exhibited significant variations, aligning with expectations. These group differences are shown in Figure 3.

**Conclusion:**

This study underscores the common occurrence of cognitive aging among the healthy population and demonstrates that MAP‐MRI is a valuable tool for assessing GM microstructural changes, particularly when comparing these changes to those observed in resilient aging populations.